# Supplementary material for: HIVprotI: an integrated web based platform for prediction and design of HIV proteins inhibitors
Source: J Cheminform. 2018 Mar 9;10:12. doi: 10.1186/s13321-018-0266-y (PMC5845081; doi:10.1186/s13321-018-0266-y)
Supplement: Supplementary file 2 — Additional file 2. Source code of HIVProtI web server. [file 13321_2018_266_MOESM2_ESM.zip › HIVprotI_Source-code/hivprotI/weka-3-6-6/doc/weka/core/Option.html]

Option


---


|  |  |  |
| --- | --- | --- |
| **Overview** \| **Package** \| **Class** \| **Tree** \| **Deprecated** \| **Index** \| **Help** \| **Weka's home** | |  |
| **PREV CLASS**   **NEXT CLASS** | **FRAMES**    **NO FRAMES**     **All Classes** |  |
| SUMMARY: NESTED \| FIELD \| CONSTR \| METHOD | DETAIL: FIELD \| CONSTR \| METHOD |  |


---


## weka.core Class Option

```
java.lang.Object
  weka.core.Option
```

**All Implemented Interfaces:**: RevisionHandler

---

``` public class Option extends java.lang.Object implements RevisionHandler ```

Class to store information about an option.

Typical usage:

`Option myOption = new Option("Uses extended mode.", "E", 0, "-E"));` 

**Version:**
:   $Revision: 1.7 $

**Author:**
:   Eibe Frank (eibe@cs.waikato.ac.nz)

---

**Constructor Summary**

`Option(java.lang.String description,
java.lang.String name,
int numArguments,
java.lang.String synopsis)`
  
          Creates new option with the given parameters.


| **Method Summary** | |
| --- | --- |
| `java.lang.String` | `description()`             Returns the option's description. |
| `java.lang.String` | `getRevision()`             Returns the revision string. |
| `java.lang.String` | `name()`             Returns the option's name. |
| `int` | `numArguments()`             Returns the option's number of arguments. |
| `java.lang.String` | `synopsis()`             Returns the option's synopsis. |

**Methods inherited from class java.lang.Object**

`equals, getClass, hashCode, notify, notifyAll, toString, wait, wait, wait`

**Constructor Detail**

### Option

```
public Option(java.lang.String description,
              java.lang.String name,
              int numArguments,
              java.lang.String synopsis)
```

:   Creates new option with the given parameters.

    **Parameters:**: `description` - the option's description: `name` - the option's name: `numArguments` - the number of arguments


**Method Detail**

### description

```
public java.lang.String description()
```

:   Returns the option's description.

    :   **Returns:**: the option's description

---


### name

```
public java.lang.String name()
```

:   Returns the option's name.

    :   **Returns:**: the option's name

---


### numArguments

```
public int numArguments()
```

:   Returns the option's number of arguments.

    :   **Returns:**: the option's number of arguments

---


### synopsis

```
public java.lang.String synopsis()
```

:   Returns the option's synopsis.

    :   **Returns:**: the option's synopsis

---


### getRevision

```
public java.lang.String getRevision()
```

:   Returns the revision string.

    :   **Specified by:**: `getRevision` in interface `RevisionHandler`
    :   **Returns:**: the revision


---


|  |  |  |
| --- | --- | --- |
| **Overview** \| **Package** \| **Class** \| **Tree** \| **Deprecated** \| **Index** \| **Help** \| **Weka's home** | |  |
| **PREV CLASS**   **NEXT CLASS** | **FRAMES**    **NO FRAMES**     **All Classes** |  |
| SUMMARY: NESTED \| FIELD \| CONSTR \| METHOD | DETAIL: FIELD \| CONSTR \| METHOD |  |


---
